# PROTOCOL: Gender‐Responsive Macro‐Level Policies and Women's Economic Empowerment in Sub‐Saharan Africa: An Evidence and Gap Map

**DOI:** 10.1002/cl2.70035

**Published:** 2025-03-22

**Authors:** David Sarfo Ameyaw, Takyiwaa Manuh, Sheila Agyemang Oppong, Clarice Panyin Nyan

**Affiliations:** ^1^ International Centre for Evaluation and Development Tema Accra Ghana

**Keywords:** evidence and gap map, gender‐responsive policies, macro‐level policies, sub‐Saharan Africa, women's economic empowerment

## Abstract

This Evidence and Gap Map (EGM) protocol aims to identify, map, and provide an overview of the existing evidence and gaps on gender‐responsive macro‐level policies and women's economic empowerment in sub‐Saharan Africa. Specifically, the EGM will: (1) identify which evidence clusters present opportunities for evidence synthesis and (2) identify the evidence gaps that require additional studies, research, and evaluations.

## 1 Background

### 1.1 Introduction

#### 1.1.1 The Problem, Condition, or Issue

Macro‐level policies play a pivotal role in shaping sub‐Saharan Africa's (SSA) trajectory and development options, and aim to foster growth, inclusion, and sustainable development (International Labour Organization 2021). They encompass a wide array of economic and social policies, spanning from fiscal, monetary, and trade policies to social protection. While these policies are presented as benefiting the whole population, there remain crucial questions regarding their impact on women's economic empowerment, inclusion, and well‐being as they concentrate on standard economic indicators like Gross Domestic Product growth, inflation rates, or balance of payments. However, they often fail to consider gender‐specific measures and interventions that are critical to advancing women's economic empowerment.

Furthermore, some policies, like export‐led growth, may inadvertently exacerbate gender disparities, concentrating women in low‐paying, insecure employment, while doing little to tackle existing care and unpaid work responsibilities that are usually borne by women and girls, based on social‐cultural norms and gender divisions of labor in households. This gender‐blind approach in macro‐level policies hinders the achievement of broader development objectives and constrains women's economic opportunities, well‐being, and agency. Thus, a comprehensive understanding of gender dynamics in national contexts is essential, necessitating informed policy adjustments and a commitment to reshaping policies and strategies for inclusive and sustainable growth through the development and use of gender‐responsive approaches and initiatives.

As noted by Duflo (2020) and Dunsch (2022) whose studies in the past have primarily focused on micro‐level interventions rather than macro‐level policies (there has also been a massive focus on micro‐level interventions such as financial assistance, including micro‐credits and capital, mentorship programs, and capacity‐building efforts toward Women's Economic Empowerment (World Economic Forum 2020). In Uganda, for instance, projects/policy interventions have also focused on vocational and life skills training programs and how they improve the lives of adolescent girls (Bandiera et al. 2020). However, the importance of macro‐level policies in shaping the broader landscape of women's economic participation is increasingly acknowledged (World Bank 2019). As a result, this evidence and gap map will specifically focus on macro‐level policies as interventions.

International frameworks, like the United Nations' Sustainable Development Goals (SDGs), emphasize the need for comprehensive macro‐level strategies to improve women's economic opportunities (Heintz 2015). The emphasis is on addressing systemic barriers and fostering an inclusive economic environment through policies that promote gender equality on a larger scale. Although this recognition is growing, there are still issues with data gaps, inconsistent reporting, and a lack of a thorough understanding of macro‐level interventions (World Economic Forum 2020). For these reasons, this evidence and gap map aims to identify the evidence and gaps in the literature and pave the way for a more comprehensive understanding of how macro‐level policies can contribute to women's empowerment.

Again efforts are being made globally to bridge these gaps and advance women's economic empowerment through macro‐level policies (OECD 2019). Governments and central banks are increasingly focusing on designing and implementing policies that create an enabling environment for women's economic participation at the broader societal level (Duflo 2012). These policies encompass fiscal, monetary, trade, social protection, and informal economy policies aimed at advancing women's opportunities. Thus, while in the past, the focus has been on micro‐level interventions for women's economic empowerment, this evidence and gap map will contribute to the emerging body of knowledge of the impact of macro‐level policies. This would provide insights into how these policies can address systemic barriers and foster an inclusive economic environment, ensuring women have equal opportunities for economic participation and contribution at a broader societal level.

#### 1.1.2 Why It Is Important to Develop the EGM

Women's economic empowerment is an important component of the SDG 5 goal of gender equality. Given that the goals intersect interventions aimed, for example, at unpaid care work, a critical barrier to women's economic empowerment and gender equality, also have the potential to affect other goals, such as SDG 2 (no poverty), SDG 3 (good health and wellbeing), SDG 4 (quality education), SDG 8 (decent work and economic growth), and SDG 10 (reduced inequality) (UN Women 2018). However, it is necessary to ensure alignment of the policies for the desired outcomes to be achieved, and decisions on which policies to implement must be based on evidence (Snilstveit et al. 2016). This EGM will present an overview of the evidence on macroeconomic policies and the Women Economic Empowerment outcomes associated with them, to aid decision‐making and help prioritize future research based on the gaps in literature.

As far as we know, there is no existing EGM on gender‐responsive macro‐level policies and women's economic empowerment outcomes, especially in sub‐Saharan Africa. An EGM that had similar women's economic empowerment outcomes but is different in scope is that of Singh et al. (2022), which focused on interventions, such as capacity building and economic support for women's empowerment. The policy section focused on both policy and institutional interventions, which does not discuss policies at the macro‐level.

Other EGMs with some women's empowerment outcomes had different scopes: one focused on the impact of infrastructure on women's empowerment and gender equality (Odei Obeng‐Amoako et al. 2023); food systems and nutrition (Moore et al. 2021); health and social care interventions to support older adults (Welch et al. 2021); and group‐based livelihood interventions on social and empowerment outcomes (Barooah et al. 2019).

Our aim is thus to develop an up‐to‐date and more thorough EGM that incorporates macro‐level policies that are gender responsive and lead to women's economic empowerment (refer to Tables 1 and 2 for intervention and outcome classification).

**Table 1:** Intervention categories and sub‐categories.

Intervention categories	Sub‐categories	Description	Examples
Macroeconomic policies		Macroeconomic policies are interventions designed to regulate an economy and achieve specific objectives. The primary goals of these policies include fostering robust economic growth, maintaining a sustainable balance of payments, ensuring low and stable inflation, and reducing unemployment. Furthermore, these policies can contribute to poverty alleviation and promote social equity, as well as help prevent recurring cycles of economic booms and recessions.	Fiscal policies Monetary policies Trade policies
	Fiscal Policies	Government's policies and measures used to manage its revenue and expenditure to achieve certain economic objectives. These policies are typically implemented through changes in taxation, government spending, revenue management, and borrowing.	Government Budget (spending‐wage bill, taxes − tax rates, exemptions, public investments − education, health and care) and infrastructure (roads, water, electricity, and energy) Gender budget initiatives and gender tracker
	Monetary policies	Interventions undertaken by the central bank or monetary authority to control and regulate the money supply, interest rates, and credit conditions in the economy and their effects, especially on the informal sector.	Interest rates, exchange rate changes, reserve rate requirements (effect on demand, prices, and informal sector enterprises and operators)
	Trade Policies	Interventions or actions taken by governments to regulate and influence international trade flows and conditions. These interventions are aimed at promoting economic growth, protecting domestic industries, and ensuring fair and balanced trade relationships with other countries.	Tariffs/Import Duties, Export subsidies Trade remedies Trade liberalization
Social Protection policies		Interventions designed to aid and support individuals and households that are vulnerable, disadvantaged, or facing social and economic risks. These interventions aim to reduce poverty, inequality, and social exclusion by providing various forms of social assistance and insurance.	
	Social housing policies	Social housing policies are initiatives implemented by governments or organizations to provide affordable housing options for individuals and families who are unable to access decent and affordable housing in the private market. These policies aim to address housing affordability, reduce homelessness, and ensure that all citizens have access to safe and stable housing.	Rental schemes, mortgage arrangements, affordable housing schemes
	Health insurance policies	Health insurance interventions involve various strategies and initiatives aimed at improving access to healthcare services and reducing financial barriers for individuals and communities. These interventions are designed to ensure that people can receive necessary medical care without facing significant out‐of‐pocket costs.	Universal health coverage, public health insurance, subsidized premiums, rural health insurance
	Childcare policies	Policies aimed at improving the accessibility, affordability, and overall quality of childcare services. These interventions recognize the importance of early childhood development and the role of high‐quality childcare in supporting children's well‐being, cognitive development, and school readiness	Early Childhood Development and Care support Childcare Subsidies and Financial Assistance Maternity and paternity leave
Informal Economy policies		Interventions implemented to address the challenges and improve the conditions of workers and businesses operating in the informal sector. The informal economy comprises economic activities that are not regulated or protected by formal labor laws, social security systems, or government oversight	Access to finance (government policies to help informal sector gain access to finance) Land titling and certification Pensions Adoption of ILO minimum social floors
	Unpaid care work policies	Government policies and measures aimed at recognizing, reducing, and redistributing the burden of unpaid care, which predominantly falls on women and girls. These interventions seek to address gender inequalities, promote gender equality, and support women's economic empowerment, and the impact of pandemics such as COVID‐19.	Public acknowledgment and support of women's care responsibilities Rebalancing care work Public financing of childcare services Investments in energy, transport, water and sanitation, access to roads (all of which reduce women's time burdens) Child benefits Double burden of care support
	Domestic work support	Encompass regulations, labor rights, and protections for domestic workers, including minimum wage laws, working hour limits, and provisions for occupational safety and health. These measures aim to ensure fair and dignified working conditions for domestic workers.	Legislation; ratification of ILO Convention 189
	Decent work policies	Policies aimed at promoting and ensuring decent working conditions, fair employment opportunities, and social protection for workers. These interventions recognize the importance of providing decent and productive work as a key driver of sustainable economic growth and social development.	Labor Rights and Protection policies Minimum Wage Policies, Social Security and Benefits Occupational Safety and Health Policies to reduce gender gap in years of education,
WEE Advocacy		Advocating for Women's Economic Empowerment at the macro‐level employing several interventions to effectively promote gender‐responsive macro‐level policies and address the specific challenges women face in the economy.	This is provided by women's rights organizations, advocates, and feminist movements
	Training and capacity building for policy makers and advocates	Providing training and resources to enhance the skills and knowledge of policy makers, advocates, and community members.	
	Policy Advocacy	Engaging with policymakers, government officials, and legislators to influence the development and implementation of gender‐responsive macro‐level policies.	
	Public Awareness Campaigns	Using various media and communication channels to raise public awareness about the importance of gender‐responsive macro‐level policies, generating support, and mobilizing public opinion.	Media campaigns Rallies
	Coalition Building	Forming alliances and partnerships with like‐minded organizations, stakeholders (IFIs, Bilateral, multilaterals), and individuals to amplify the advocacy message and increase its impact.	

**Table 2:** Outcome categories and sub‐categories.

Outcome category	Sub‐categories	Definitions/Examples
Women's economic opportunities		Focuses on enabling environment that encourages women's economic participation.
	Employment	Employed, Unemployed, employment status, employment opportunities, formal and informal work.
	Income (earnings)	Salary, wage, earnings, entrepreneurial profits, gender wage gap‐difference in earnings between women and men in the labor market.
	Access to credit and financial services	Women's ability to access financial resources, including loans, credit, and savings accounts. Proportion of women with access to formal financial services and their utilization of these services.
	Business ownership and entrepreneurship	Women who own businesses or are engaged in entrepreneurial activities. Women's participation in economic ventures and their ability to create and grow enterprises.
	Education and skills attainment (including digital technology literacy/inclusiveness)	Women's educational attainment levels, including primary, secondary, and tertiary education. Gender gap in educational enrollment rates and the availability of skill development programs, including digital technology targeted at women. E.g. Literacy rate, digital literacy.
	Women's work productivity	The efficiency and effectiveness with which an individual, team, or organization accomplishes tasks and produces desired outcomes in a given period.
Agency		Ability of women to make choices, exercise control over their lives, and participate actively in social, economic, and political spheres. It encompasses their capacity to make decisions regarding their education, career, health, relationships, and overall well‐being. Women's agency is a fundamental aspect of gender equality and women's empowerment.
	Women in leadership positions	Women in leadership roles within the public and private sectors. For instance, women in managerial positions, executive roles, and on corporate boards. Increasing this proportion reflects improved opportunities for women to influence decision‐making and shape economic policies.
	Control over productive resources	Women's access to and control over productive resources, such as land, property, technology, and agricultural inputs. Ownership rights, secure land tenure, and equal access to resources necessary for economic activities.
	Women's participation in decision‐making	Agency, Women involved in economic decision‐making processes at the household, community, and national levels. Women's participation in decision‐making forums, their access to information, and their influence over economic choices. Financial independence (or dependence on others financially).
Well‐being		Well‐being deals with the quality of life and the capacity of individuals (women and girls) to make meaningful contributions.
	Physical well‐being	Physical health and functioning, including factors such as nutrition, exercise, sleep, and freedom from illness or disease and violence.
	Mental well‐being	Emotional and psychological state. Having a positive sense of self, experiencing positive emotions, managing stress effectively, and the ability to cope with challenges and setbacks.
	Social well‐being	Relationships and social connections. Having supportive and meaningful relationships with family, friends, and communities. Social well‐being encompasses feelings of belonging, inclusion, and social support. Risk of gender‐based violence.
Process insights		Process evaluations/studies that delve into the workings of interventions, exploring both their theoretical underpinnings, such as the theory of change, as well as practical aspects, including design, costs, and implementation considerations.
	Intervention details	Intervention design features (i.e., what details of what is being delivered)
	Implementation	Barriers and facilitators (i.e., what helps and/or hinders when delivering an intervention in practice)
	Theory of change	Theory of change for the intervention (i.e., how the intervention achieves its aim(s) in theory)
	Cost	Cost analysis (CA), Cost‐Benefit‐Analysis (CBA), and cost data (i.e., if the study contained any information about the cost of the intervention or featured any types of cost analysis).

### 1.2 Conceptual Framework

**Figure 1:**
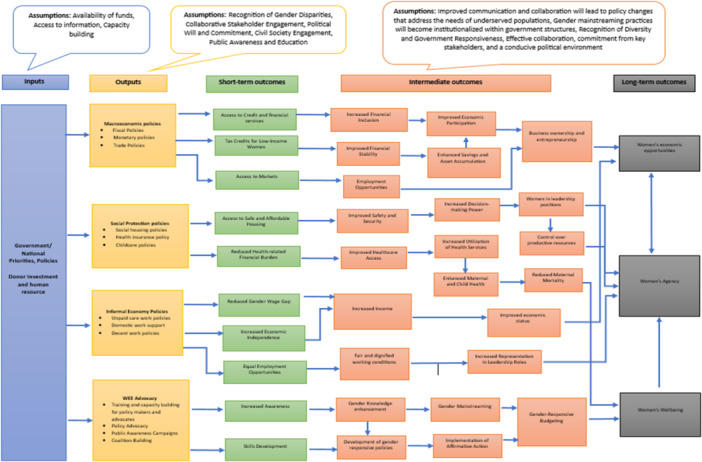
Conceptual framework.

The conceptual framework (Figure 1) of this EGM explains how gender responsive macro‐level policies are hypothesized to affect the three main outcomes of interest: women's economic opportunities, agency, and well‐being. These outcomes could be either short‐term or intermediate outcomes, which eventually result in long‐term outcomes. Four gender‐responsive domains and women's economic empowerment interventions are explained in this framework. They include macroeconomic policies where interventions, such as fiscal policies, monetary policies, and trade policies are considered; social protection policies where interventions, such as social housing, health insurance, and child care policies are examined; informal economy policies where interventions on unpaid care work, domestic work support, and decent work policies are considered; and women's economic empowerment advocacy where interventions, such as training and capacity‐building for policy‐makers and advocates, policy advocacy, public awareness campaigns, and coalition building are explored.

Macroeconomic policies, such as fiscal policies, monetary policies, and trade policies, comprise various interventions. These interventions yield short‐term outcomes, including increased access to credit and financial services, tax credits for low‐income women, and improved access to markets. These measures could result in lower interest rates and targeted credit easing, which ultimately may enhance access to financing for women entrepreneurs and businesses, thus promoting financial inclusion. Targeted tax credits for low‐income women or families provide immediate financial relief, reducing economic disparities and supporting women's economic well‐being. Additionally, trade policies that facilitate market access for products and services produced by women‐owned businesses can lead to an immediate expansion of their customer base, resulting in increased sales and revenue (Dela Cruz et al. 2023). Consequently, these short‐term outcomes may contribute to financial inclusion, financial stability, and the creation of employment opportunities for women. As a result, women gain access to financial services, enabling them to actively participate in economic activities and increase their access to credit. This, in turn, allows women to start or expand their small businesses, enhancing their economic contribution. Simultaneously, these interventions also foster financial stability by promoting savings and asset accumulation for women. They can securely save money and accumulate assets, thus improving financial stability. In the long term, the intermediate outcomes of employment opportunities, business ownership and entrepreneurship resulting from macroeconomic policy interventions lead to enhanced economic opportunities, agency, and well‐being for women (Cabeza‐García et al. 2019).

Social protection policies interventions include social housing policies, health insurance policies, and childcare policies. The short‐term outcome of this intervention includes improved access to safe and affordable housing for women and their families. By providing secure and affordable housing options, the risk of homelessness or unstable living conditions for women is likely to reduce. Additionally, health insurance coverage may help alleviate the financial burden associated with medical expenses for women, leading to reduced health‐related financial stress. With reduced out‐of‐pocket costs for healthcare, women can allocate their resources to other economic priorities. Access to health insurance could possibly enable women to seek timely medical care and preventive services, resulting in improved health outcomes and overall well‐being. This, in turn, positively impacts their ability to engage in economic activities (OECD 2019).

Furthermore, improved safety and security could be achieved through access to safe housing. By reducing women's vulnerability to violence, harassment, and other security risks, affordable housing in secure neighborhoods contributes to the personal safety of women and their families. Additionally, improved healthcare access may provide women and girls with better access to healthcare, which is crucial for building resilience against future crises. Consequently, this leads to an increase in decision‐making power for women, greater utilization of health services, and enhancement of maternal and child health. These outcomes also contribute to women assuming leadership positions, gaining control over productive resources, and reducing maternal mortality rates due to improved access to healthcare services. Ultimately, these achievements have long‐term effects on women's economic opportunities, agency, and well‐being.

Policies addressing the informal economy include measures, such as unpaid care work policies, domestic work support, and decent work policies. These interventions might have various positive outcomes for women in the short term, such as reducing the gender wage gap, increasing economic independence, and providing equal employment opportunities. By addressing unpaid care work, these policies could promote equal participation of women in the workforce, ultimately leading to a narrower gender wage gap. Additionally, improved working conditions and fair compensation may well enhance the economic independence of women engaged in domestic work. In the short term, these interventions could also result in better access to equal employment opportunities, ensuring that women have the same chances as men to secure jobs and advance in their careers. As a result, women may experience increased income and fair and dignified working conditions, thereby improving their status in society. These positive outcomes further contribute to improved economic status and increased representation of women in leadership roles as intermediate outcomes. In the long term, these interventions possibly will lead to greater agency, increased economic opportunities for women, and overall well‐being.

Women's economic empowerment advocacy interventions include training and capacity‐building for policy makers and advocates, policy advocacy, public awareness campaigns, and coalition building. When these interventions are implemented, they may perhaps result in increased awareness and skills development for policy makers and advocates in the short‐term. Advocacy efforts could also raise awareness and understanding of the importance of women's economic empowerment among the general public, policymakers, and businesses. Successful advocacy may lead to increased media coverage, which brings attention to the challenges women face in the economic sphere and highlights success stories. These short‐term outcomes may contribute to increased awareness of existing gender disparities and the need for policies that promote women's economic empowerment. This, in turn, leads to discussions and debates within legislative bodies or policy forums regarding the need for specific policies addressing gender‐based economic inequalities. As a result, there might be an enhancement of gender knowledge, development of gender‐responsive policies, gender mainstreaming, implementation of affirmative action, and ultimately, gender‐responsive budgeting as intermediate outcomes. In the long‐term, women's economic empowerment advocacy interventions contribute to the well‐being, agency, and economic empowerment of women.

## 2 Objectives

The aim of this EGM is to identify, map, and provide an overview of the existing evidence and gaps on gender‐responsive macro‐level policies and women's economic empowerment in sub‐Saharan Africa. The EGM will specifically:

1. Identify which evidence clusters present opportunities for evidence synthesis.
2. Identify the evidence gaps that require additional studies, research, and evaluations.

## 3 Methods

Our EGM will follow the Campbell Collaboration's guidance for creating evidence and gap maps (White et al. 2020).

### 3.1 Defining an EGM and Purpose

An EGM is a user‐friendly approach that shows the extent to which studies have been done on a particular topic. White et al. (2020) define EGMs as “systematic evidence synthesis product which displays the available evidence relevant to a specific research question.” The production of an EGM follows a comprehensive search, screening based on the criteria developed to include or exclude studies, systematic coding, analysis, and reporting. Usually, findings from EGM are presented in a two‐dimensional matrix with interventions as row headings and outcomes as column headings (White et al. 2020).

Our EGM will serve as a platform for evidence on the relationship between gender responsive macro‐level policies and women's economic empowerment. Further, our EGM will highlight the gaps in macro‐level policy interventions, which will be used by all stakeholders, including researchers, government bodies, policy makers, and practitioners.

### 3.2 Scope of EGM

To produce our EGM, we will use the population, intervention, comparison, outcome, and study design (PICOS) framework, which covers studies on interventions for women and girls. Studies (evaluation, experimental, quasi‐experimental, nonexperimental, meta‐analyses, and systematic reviews) will be included. The interventions of the EGM will have a focus on macro‐level policies that are gender responsive (see Table 1), focusing on women and girls with the main thematic domain of women's economic empowerment, including women's economic opportunities, agency, and wellbeing (see Table 2). We will include in our EGM studies published in the year 1990 and beyond, to be able to capture and include relevant studies in our scope.

#### 3.2.1 Population

The population of interest for our EGM will include women and girls of all ages in sub‐Saharan Africa. Sub‐Saharan Africa is defined by the World Bank as the varied region of the continent that includes low‐, lower‐, upper‐, and high‐income nations. The region faces several development‐related obstacles generally (World Bank 2023). The terms “women” and “girls” as suggested in this study refer to the biological construct of females of all ages.

#### 3.2.2 Intervention

The intervention for this EGM is macro‐level policies, which refer to large‐scale, overarching strategies and initiatives implemented by governments or other governing bodies to influence and regulate the overall economic, social, and political conditions within a society. These policies operate at a broad level, impacting entire sectors of the economy or society rather than individual businesses or individuals. In the context of WEE, macro‐level policies would include government actions and regulations that aim to address systemic issues and promote gender equality on a societal scale. These policies may span various domains, including fiscal policy (government taxation and spending), monetary policy (control of the money supply and interest rates), trade policies, social protection policies, and policies affecting the informal economy.

#### 3.2.3 Comparison

Our EGM will not include a comparison group. However, research such as impact studies and comparison studies, which compare various situations to identify patterns, would be included.

#### 3.2.4 Outcomes

The outcome of this EGM is women's economic empowerment categorized as women's economic opportunities, agency, and well‐being (see Table 2 for outcome categories and sub‐categories).

#### 3.2.5 Study Design

We will include both published and unpublished studies, reports, and reviews with the following study designs:

- Experimental study.
- Nonexperimental (quasi‐experiment, descriptive, and regression‐based study).
- Case study and other qualitative studies.
- Modeling studies.
- Systematic reviews.
- Scoping reviews.

Additionally, effectiveness studies (impact evaluations/experimental studies); modeling studies (for models based on primary data and not hypothetical values); process evaluations; summative evaluations; qualitative assessments; and analytical frameworks will be included. Given the lack of available literature on the topic, including a wide range of study designs in this EGM will be beneficial: (i) to include all relevant studies; and (ii) to assess the amount of evidence across various study designs. Research methodologies (e.g., exploratory, narrative, deductive, inductive, ethnographic, and grounded theory approaches) should be clearly described in eligible qualitative studies. We will also include systematic and scoping reviews of the eligible primary studies, peer‐reviewed papers, preprints, reports, discussion, and working papers.

#### 3.2.6 How We Plan to Handle Adverse Outcomes

To prevent the reporting of only one side of the effects of interventions (White et al. 2020), this EGM will take into account studies where interventions for gender‐responsive macro‐level policies produced adverse outcomes. Since the outcomes in the EGM are neutral, adverse outcomes will be coded in the EGM under the outcome they fall under, just like in the case of positive outcomes.

### 3.3 Other Inclusion and Exclusion Criteria for Eligible Studies

Based on the defined population, intervention, outcome, and study design (PICOS) for this EGM, we will assess the eligibility of the included studies (i.e., published and unpublished) (White et al. 2018).

#### 3.3.1 Type and Status of Studies

We will include both completed and ongoing studies. For ongoing studies, we will include available protocols when available. This EGM will include all kinds of studies that applied all kinds of study designs, such as quantitative, qualitative, or mixed methods.

#### 3.3.2 Geographical Location

We will include studies conducted in sub‐Saharan Africa. Eligible studies for our EGM will be from the sub‐Saharan Africa region regardless of the study settings. By following the World Bank's country classification by income: low‐income economies, lower‐middle‐income economies, upper‐middle‐income economies, and high‐income economies (World Bank 2023), all studies in this category under the sub‐Saharan Africa region will be included.

#### 3.3.3 Timeframe

Eligible studies for our EGM will include studies published/reported in the years from 1990 and beyond. This timeframe was chosen because of the increased focus on women's economic empowerment during the 1990s, particularly highlighted by the United Nations' SDG 5: Achieve gender equality and empower all women and girls. By selecting this specific timeframe, we aim to comprehensively capture all relevant research related to women empowerment in sub‐Saharan Africa within our search results (UN Women 2018).

#### 3.3.4 Language

Studies published/written in English and French will be included, though the search terms will be developed in English only.

### 3.4 Search Strategy and Search Terms

#### 3.4.1 Search Strategy

Our EGM will employ an information specialist to assist with the search strategy for identifying all relevant studies, whether published or unpublished. We will use Cab Abstracts, Scopus, Web of Science, and Dimensions as our academic database. In addition, the OpenAlex in EPPI‐reviewer software will be utilized to search for any additional eligible papers. Our focus will be on studies published in both English and French.

We will further conduct a gray literature search from 22 organizational websites and electronic databases:

- EPPI knowledge library.
- Social Science Research Network.
- Centre for Strategic and International Studies.
- Abdul Latif Jameel Poverty Action Lab.
- Innovations for Poverty Action (IPA).
- UN Women.
- International Centre for Research on Women.
- Engender Impact.
- Econlit.
- National Bureau of Economic Research (NBER).
- USAID‐ Development Experience Clearinghouse (DEC).
- World Bank Economic Review.
- World Bank documents and reports.
- The Cochrane Central Register of Controlled Trials (CENTRAL).
- The Campbell Library.
- The Cochrane Library.
- Social Science Research Network (SSRN).
- World Bank – Independent Evaluation Group (IEG).
- African Development Bank – Evaluation reports.
- Asian Development Bank – Evaluation resources.
- Google Scholar.
- IDEAS RePEc.
- Gender at Work.

We will further conduct hand searches in these journals:

- Journal of Development Economics.
- Journal of International Development.
- European Journal of Development Research.
- Asian Journal of Women's Studies.
- International Journal of Women's Empowerment.
- Journal of Women's Empowerment and Strategy.
- Social Indicators Research.
- World Development.
- Development and Change.
- Gender and Development.
- Feminist Economics.
- IDS Bulletin.
- Sabinet African Journals.
- Policy Commons.

To discover additional primary studies and reviews that may not have been identified in our initial search but are still pertinent to our EGM, we will utilize the backward‐track citations approach. To ensure comprehensive coverage of relevant evidence in the EGM, we will prioritize authors who are frequently cited in papers within the subject matter field. Additionally, we will search registries or repositories for completed and ongoing trials, reviews, and EGMs.

Additionally, evaluation reports, university theses, and other gray literature will provide supplementary studies. A valuable source of information for potential studies to incorporate into our EGM will be the relevant EGMs already completed by institutions, such as Campbell Collaboration and 3ie.

Every eligible manuscript will be uploaded into the EPPI‐Reviewer program and converted into a Research Information System (RIS) (Thomas and Brunton 2010). To fulfill reporting requirements, we will keep a record of our literature search activities.

#### 3.4.2 Search Terms

We will develop search terms based on our PICOS framework, where we will develop four search facets: population, intervention, and outcome, and may include the study design. These will be retrieved from published documents from electronic databases, and others from gray sources based on relevant key terms from studies.

Our search terms for our intervention category will focus on macroeconomic policies (fiscal policies, monetary policies, and trade policies), social protection policies (social housing policies, health insurance interventions and childcare support), policies on informal economy (unpaid care work, domestic work, decent work support policies) and policies on advocacy toward women's economic empowerment. We will also make use of the Boolean operators and wildcards (see Appendix 1, e.g., of search terms).

### 3.5 Screening

Screening of data will be done using EPPI‐reviewer software. Using the EPPI‐Reviewer software, all duplicates will be identified and removed before reviewing the potential studies or papers. We will utilize a machine learning model to assist in ranking the identified articles based on their relevance and priority, in accordance with the eligibility requirements for the proposed EGM review. This will help expedite the screening process.

**Figure 2:**
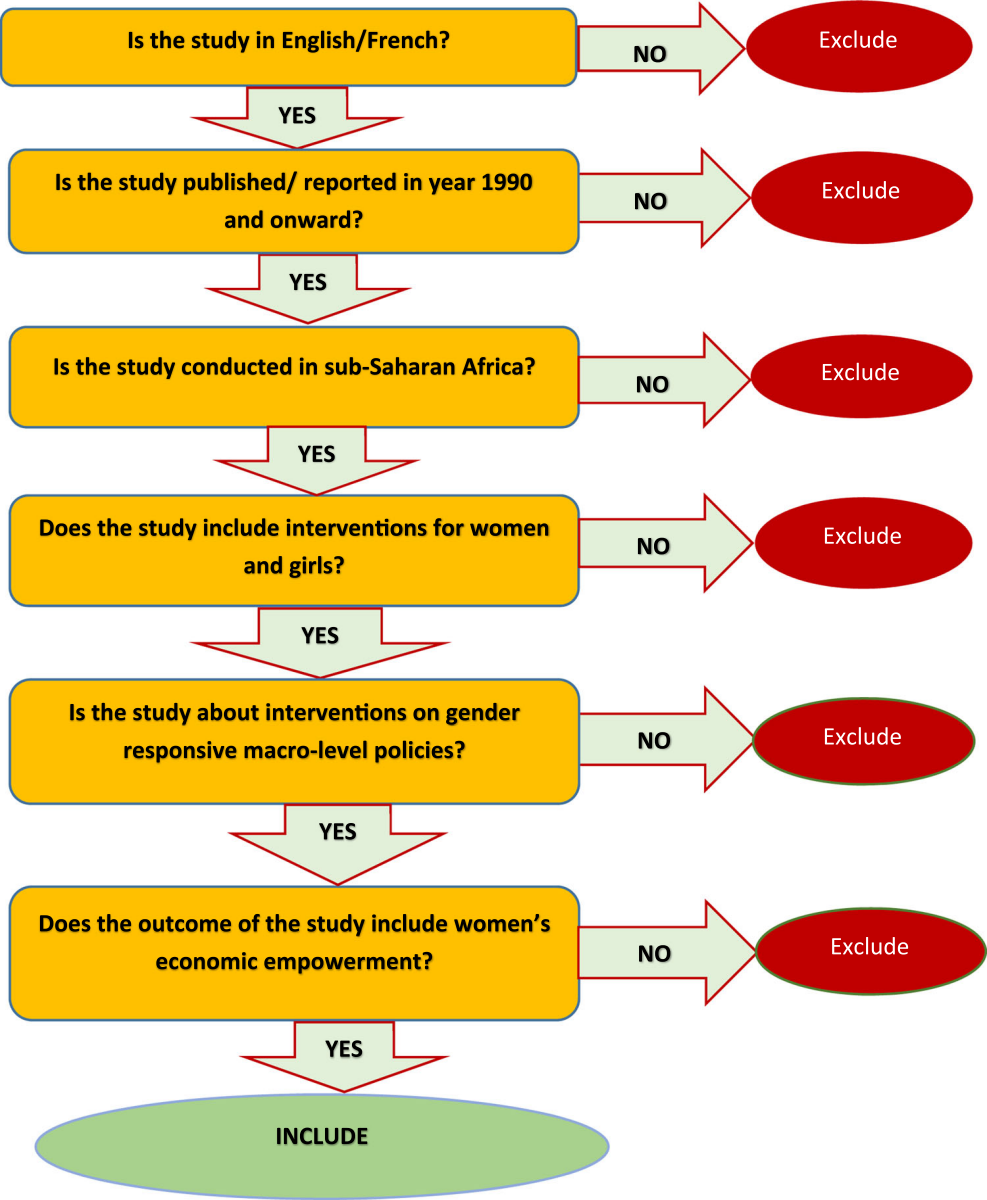
Screening tool to assess eligible studies.

There will be two stages of eligibility screening for the EGM, which includes both published and unpublished studies. The studies will initially undergo a review of their title and abstract in accordance with the eligibility criteria, which includes both inclusion and exclusion criteria (see Figure 2 below). Following the title and abstract screening, a full‐text screening will be conducted to determine which research should be included. Each study will be independently reviewed by two reviewers. In cases where the two reviewers cannot reach a consensus on whether to include a paper in the EGM, they will consult with each other, and if necessary, a third reviewer will independently conduct the screening to resolve any disagreements.

### 3.6 Data Extraction (Coding) and Analysis

#### 3.6.1 Coding

A data extraction form will be used to extract data from all types of included studies. This form will display information such as the region, country, language, population, study status, intervention, outcome, study design, and evaluation type. For data management, analysis, and coding, we will use EPPI‐Reviewer (Thomas and Brunton 2010). Duplicate data will be retrieved for comparison, and each study or paper will be coded in EPPI‐Reviewer according to a predetermined form (refer to Appendix 2 for the Coding form).

#### 3.6.2 Data Analysis

Before analysis, the data will be cleaned to remove errors, duplicates, inconsistencies, outliers, and missing studies from the data set. We will use univariate analysis, for instance, to identify outliers in the code. We will then conduct an initial pilot test of our data collection form using EPPI Reviewer. Following the pilot test, we will proceed to individually extract and code the data. To ensure accuracy, a second reviewer will validate a sample of the data extraction and coding. For systematic and scoping reviews, data extraction will be conducted based on the eligible included studies.

As with previous EGMs, we will include the most recent open‐access publication in the EGM when multiple publications are found on a single study, such as a working paper and a published paper (Malhotra et al. 2021). If a study reports on several interventions, outcomes, or study designs, it will be coded for the interventions, outcomes, or study designs, but it will be viewed as a single entry in the EGM. Similarly, we will only consider the interventions that are relevant to our EGM if a single research has several interventions, some of which are not eligible for our EGM. On the other hand, primary research that meets the criteria for our EGM and appears in a systematic review will be counted as one analysis unit.

The distribution and features of the studies in the EGM will be described using summary statistics such as percentages. The results will be presented in graphs, tables, and cross tabulations of the intervention against outcome categories, regions, and research design in a narrative report for the EGM. Subsequently, the findings will be compiled and integrated to identify evidentiary gaps and clusters. The EGM will be created using the EPPI‐Reviewer software based on the data submitted into the EPPI‐Reviewer. The software will also be used to generate descriptive statistics for the technical report (Thomas and Brunton 2010).

#### 3.6.3 Presentation of EGM

Our EGM will be organized as a matrix with easily navigable search filters. The main dimensions of the matrix will be interventions and outcomes. We will work closely with important partners like the Campbell Collaboration and the Hewlett Foundation to jointly define the framework for the map, which will include heading titles, filters, and row headings for intervention categories and subcategories that are aligned with outcome domains and subdomains that serve as column headings. Relevant studies will be documented within the matrix cells that correspond to combinations of interventions and outcomes. Inspired by the methodology presented by White et al. (2018) and Moore et al. (2021), the matrix will have searchable filters covering intervention specifics, research design, study status (finished and ongoing), country and area, and location to improve usability.

#### 3.6.4 Critical Quality Appraisal of Studies

We will use the AMSTAR 2 tool (Shea et al. 2017) to evaluate the methodological quality of systematic reviews. We may assess the quality of the primary research due to the tight time frame for the EGM. However, as filters for presenting the EGM, we will categorize and report the study designs employed by the qualifying studies (White et al. 2020).

### 3.7 Stakeholder Engagement

- Development practitioners: will be consulted to define our PICOS as well as use findings of the EGM to prioritize their funding decisions.
- Policy‐makers: will be consulted to define our PICOS as well as use and disseminate the results of the findings of the EGM.
- Academicians: will be consulted on the literature list to ensure that we do not miss relevant studies, as well as use the findings of the EGM to do more research on climate change adaptation that has an evidence gap.
- International Centre for Evaluation and Development (ICED) board members: make sure the EGM is conducted as planned.
- ICED Research team: conduct the EGM and disseminate the main findings of the research through various channels.
- Hewlett Foundation: provide funding to undertake the EGM; will also be consulted to define our PICOS; use findings of the EGM to prioritize their funding decisions; and disseminate the results of the EGM.
- Campbell Collaboration and Cochrane: Publishing and disseminating the EGM. We will collaborate closely with the Campbell Collaboration throughout the entire process of conceptualizing, designing, and producing the EGM. The EGM will be a public good and will be published in an open‐access journal, ensuring that it is accessible to everyone. Our goal is for the findings of our EGM to influence future decision‐making on research and funding priorities related to gender‐responsive macro‐level policies and women's economic empowerment. Specifically, we will examine agency, well‐being, and the outcomes of women's economic empowerment.

To determine the scope and structure required for the map, we conducted initial discussions with various stakeholders. This included members of the Hewlett Foundation's Gender Equity and Governance team, as well as their grantees and partners. We also engaged with Afrobarometer, the Center for Democratic Development Ghana, experts from the African Population Health Research Council, some researchers from the University of Ghana, the STAR Ghana Foundation, Women in Informal Employment: Globalizing and Organizing (WIEGO), and a few representatives from the Ministry of Finance and the Ministry of Gender, Children, and Social Protection. These meetings involved academics, researchers, and decision‐makers.

These individuals will be consulted again for feedback on the updated framework, preliminary findings, and draft map as the evidence and gap map is developed. Additionally, to gather input from a diverse range of stakeholders interested in women's empowerment, we will share the draft map and preliminary findings with them.

### 3.8 Plans to Update EGM

The proposed EGM will be updated every 2 years based on the availability of funds.

### 3.9 Sources of Technical and Financial Support

The Campbell Collaboration will work with us to develop this EGM. Campbell Collaboration has a proven record of producing EGM and increasing EGM capacity. The Hewlett Foundation will provide financial assistance and technical advice from the funder's perspective to produce this EGM, while the Campbell Collaboration will provide technological backstopping.

## Author Contributions

- **Content expertise**
  David Ameyaw is the CEO of the International Centre for Evaluation and Development (ICED) and the PI for this EGM. He has robust experience in impact evaluation, project design and management and EGMs. He also has a strong research background.
  Takyiwaa Manuh is an Emerita Professor at the University of Ghana and the gender specialist of this EGM. She brings on board her vast experience in gender related issues.
  Sheila Agyemang Oppong is a development economist specialist at ICED. She brings on board her interest in economic and international development, EGMs, and issues on women's economic empowerment and gender equality.
  Clarice Panyin Nyan is a PhD candidate at the University of Ghana. She has expertise in project management, evidence synthesis and monitoring and evaluation.
- **EGM methods expertise**
  David Ameyaw, Takyiwaa Manuh, Sheila Agyemang Oppong, and Clarice Panyin Nyan have experience in developing EGMs from previously coauthored EGMs.
- **Information retrieval expertise**
  Rodney Malesi, a senior librarian affiliated with the United States International University, will be tasked with the retrieval of relevant studies for the EGM. Rodney is an expert in literature retrieval and has worked on various systematic reviews and EGMs. He will be supported by the authors.
- **Coding and data extraction**
  Sheila Agyemang Oppong and Clarice Panyin Nyan will take the lead in all coding and data extraction activities.
- **Statistical analysis**

All the team members have the prerequisite educational and professional background necessary to conduct statistical analysis and generate meaningful reports.

## Conflicts of Interest

The authors declare no conflicts of interest.

## Sources of Support

### Internal sources

None.

### External sources

This EGM is supported by the Hewlett Foundation.

## Supporting information

Supporting information.
