# Supplementary material for: PROTOCOL: Gender‐Responsive Macro‐Level Policies and Women's Economic Empowerment in Sub‐Saharan Africa: An Evidence and Gap Map
Source: Campbell Syst Rev. 2025 Mar 22;21(2):e70035. doi: 10.1002/cl2.70035 (PMC11929552; doi:10.1002/cl2.70035)
Supplement: Supplementary file 1 — Supporting information. [file CL2-21-e70035-s001.docx]

**Appendices**

**Appendix 1: Search terms**

| **Category (All Fields)** |  | Search Terms |
| --- | --- | --- |
| 1. **Population** | 1  SSA | (“Angola” OR “Burundi” OR “Central African Republic” OR “Chad” OR “Democratic Republic of Congo” OR “Republic of Congo” OR “Rwanda” OR “Comoros” OR “Eritrea” OR “Ethiopia” OR “Kenya” OR “Madagascar” OR “Mauritius” OR “Seychelles” OR “Somalia” OR “South Sudan” OR “Sudan” OR “Tanzania” OR “Uganda” OR “Botswana” OR “Eswatini” OR “Swaziland” OR “Lesotho” OR “Malawi” OR “Mozambique” OR “Namibia” OR “South Africa” OR “Zambia” OR “Zimbabwe” OR “Benin” OR “Burkina Faso” OR “Cabo Verde” OR “Cape Verde” OR “Cameroon” OR “Cote d'Ivoire” OR “Ivory Coast” OR “Equatorial Guinea” OR “Gabon” OR “Gambia” OR “Ghana” OR “Gold Coast” OR “Guinea” OR “Guinea Bissau” OR “Portuguese Guinea” OR “Liberia” OR “Mali” OR “Mauritania” OR “Niger” OR “Nigeria” OR “Sao Tome and Principe” OR “Senegal” OR “Sierra Leone” OR “Togo” OR “africa south of the sahara” OR “sub-saharan Africa” OR “subsaharan Africa” OR “africa central” OR “central Africa” OR “africa southern” OR “southern africa” OR “africa eastern” OR “east Africa” OR “eastern Africa” OR “africa western” OR “west Africa” OR “western Africa” OR “Africa”) AND (“woman” OR “women" OR "girl*” OR “transgender” OR “female”) |
|  | 2  Target population |  |
| **Population**  (All terms and fields) | **3** | (#1 AND #2) |
| **2 Interventions** | **4**  **Fiscal policies** | “gender-based budgeting” OR “gender” AND (“budget” OR “fiscal” OR “tax” OR “macro”) |
|  | 5  **Monetary policies** | “gender” AND (“interest rates ” OR “exchange rate” OR “reserve rate requirements” OR “monetary policy”) |
|  | 6  Trade policies | “gender” AND (“tariffs dut*” OR “import dut*” OR “export subsidies” OR “trade remedies” OR “trade liberalization”) |
|  | 7  **Social housing policies** | ((“social housing” OR “housing” OR “Low-cost housing” OR “public housing” OR “rental” OR “mortgage” OR “affordable housing” OR “social protection”) AND (“polic*” OR “Schemes” OR “women”)) |
|  | 8  Health insurance policies | ((“universal” OR “national” OR “public” OR “rural” OR “maternal” OR “maternity”) AND (“health insurance policies” OR “gender”)) |
|  | 9  **Childcare policies** | TS=((“early childhood” OR “childcare” OR "school childcare" OR “maternity leave” OR “paternity leave”) AND (“polic*” OR “services” OR “subsidi*” OR “financial Assistance” OR “financial support” OR “cash transfer” OR “voucher”)) |
|  | **10**  **Unpaid care work policies** | ALL=((“compensation” OR “unpaid care” OR “flexible work agreement*” OR “pension” OR “care support” OR “transport” OR “water” OR “sanitation” OR “cash transfer” OR “care work” OR “social transfer*” OR “inclusion") AND ("polici*") AND ("wom?n" OR “gender”)) |
|  | **11**  **Domestic and decent work policies** | ((“polic?”) OR (“social security benefits”)) AND (“labo?r” OR “occupational health safety” OR “working hour limit?” OR “domestic work” OR “dignified work conditions” OR “female worker protection” OR “minimum wage?”) |
|  | **12**  **Training and capacity building for policy makers and advocates** | ((“training” OR “capacity building” OR “capacity development” OR “capacity strengthening” OR “domestic workers protection”) AND (“policy mak*” OR “CSOs” OR “media” OR “grassroot movement” OR “gender”)) |
|  | **13 Policy advocacy and**  **Public Awareness Campaigns** | ((“awareness campaigns” OR “ policy education”) AND (“public”)) OR ((“gender responsive” OR “equal participation”) AND (“policy”)) |
|  | **14**  **Coalition Building** | (“Alliance” OR “Partnership formation” OR “opinion mobilization”) AND (“Coalition building” OR “Collaboration”) |
| **Intervention**  **(All terms and fields)** | 15 | **(#4 OR #5 OR #6 OR #7 OR #8 OR #9 OR #10 OR #11 OR #12 OR #13 OR #14)** |
| 1. **Outcome** | 16  **Women's economic opportunities** | ((“paid” OR “maternal” OR “status” OR “opportunities”) AND (“employment”)) OR ((“formal” OR “informal”) AND (“work”)) OR “income” OR “salary” OR “wage” OR “earnings” OR “entrepreneurial profits” OR “gender wage gap” OR ((“credit” OR “financial services” OR “financial resource*” OR “microcredit” OR “loan” OR “savings account”) AND (“access”)) OR “business ownership” OR “entrepreneurship” OR “skills attainment” OR “digital literacy” OR ((“productivity” OR “financial protection”) AND (“wom?n”)) |
|  | **17**  **Agency** | ((“agency” OR leadership”) AND (wom?n)) OR “access to information” OR “access to resources” OR “Control over productive resources” OR “ownership rights” OR “mobility freedom” OR “exposure to media” OR “sexual empowerment” |
|  | **18**  **Wellbeing** | ((“wellbeing”) AND (“physical” OR “social” OR “mental”)) OR ((wom?n) AND (“fertility” OR “mental health” OR “healthcare access”)) |
|  | **19**  **Process insights** | “theory of change” OR “logic model” OR “process evaluation” OR “formative evaluations” OR “barriers to participation” OR “cost analysis” OR “cost benefit analysis” |
| **Outcome**  (All terms and fields) | **20** | **(#16 OR #17 OR #18 OR #19)** |
| **Study design** | **21** | “case study” OR “quantitative synthesis” OR “mixed method” “qualitative method” OR “systematic review” OR “scoping reviews” OR “summative evaluation” OR “modeling” OR “process evaluation” OR “randomized control trials" OR “regression discontinuity” OR “propensity score” OR “comparison group” OR “control group” OR “instrumental variable” OR “experimental study” OR “quasi-experiment” OR “meta-analysis” OR “descriptive” OR “regression-based study” OR “Non-experimental” OR “experimental” |
| **Combined search** | **22**  **(#3 AND #15 AND #20)** | **(#1 OR #2) AND (#4 OR #5 #6 OR #7 OR #8 OR #9 OR #10 OR #11 OR #12 OR #13 OR #14) AND (#16 OR #17 OR #18 OR #19) AND #20** |

| **Appendix 2: Coding Form** | | |
| --- | --- | --- |
|  | **Parent code** | **Child code** |
| **Language** | English |  |
|  | French |  |
| **Region** | Central Africa |  |
|  | Eastern Africa |  |
|  | Western Africa |  |
|  | Southern Africa |  |
| **Country** | Angola |  |
|  | Burundi |  |
|  | Central African Republic |  |
|  | Chad |  |
|  | Democratic Republic of Congo |  |
|  | Republic of Congo |  |
|  | Rwanda |  |
|  | Comoros |  |
|  | Eritrea |  |
|  | Ethiopia |  |
|  | Kenya |  |
|  | Madagascar |  |
|  | Mauritius |  |
|  | Seychelles |  |
|  | Somalia |  |
|  | South Sudan |  |
|  | Sudan |  |
|  | Tanzania |  |
|  | Uganda |  |
|  | Botswana |  |
|  | Eswatini (Formerly Known as Swaziland) |  |
|  | Lesotho |  |
|  | Malawi |  |
|  | Mozambique |  |
|  | Namibia |  |
|  | South Africa |  |
|  | Zambia |  |
|  | Zimbabwe |  |
|  | Benin |  |
|  | Burkina Faso |  |
|  | Cabo Verde |  |
|  | Cameroon |  |
|  | Cote d'Ivoire |  |
|  | Equatorial Guinea |  |
|  | Gabon |  |
|  | Gambia, The |  |
|  | Ghana |  |
|  | Guinea |  |
|  | Guinea-Bissau |  |
|  | Liberia |  |
|  | Mali |  |
|  | Mauritania |  |
|  | Niger |  |
|  | Nigeria |  |
|  | Sao Tome and Principe |  |
|  | Senegal |  |
|  | Sierra Leone |  |
|  | Togo |  |
| **Study status** | Completed |  |
|  | Ongoing |  |
| **Study population** | Women |  |
|  | Girls |  |
| **Intervention** | **Macroeconomic policies** | Fiscal Policies |
|  |  | Monetary policies |
|  |  | Trade Policies |
|  | **Social Protection policies** | Social housing policies |
|  |  | Health insurance policies |
|  |  | Childcare policies |
|  | **Informal Economy Policies** | Unpaid care work policies |
|  |  | Domestic work support |
|  |  | Decent work policies |
|  | **WEE Advocacy** | Training and capacity building for policy makers and advocates |
|  |  | Policy Advocacy |
|  |  | Public Awareness Campaigns |
|  |  | Coalition Building |
| **Outcome** | **Women's economic opportunities** | Employment |
|  |  | Income (earnings) |
|  |  | Access to credit and financial services |
|  |  | Business ownership and entrepreneurship |
|  |  | Education and skills attainment (including digital technology literacy/inclusiveness) |
|  |  | Women’s work productivity |
|  | **Agency** | Women in leadership positions |
|  |  | Control over productive resources |
|  |  | Women’s participation in decision-making |
|  | **Wellbeing** | Physical well-being |
|  |  | Mental well-being |
|  |  | Social well-being |
|  | **Process Insight** | Intervention details |
|  |  | Implementation |
|  |  | Theory of change |
|  |  | Cost |
| **Study design** | **Experimental** | Experimental study |
|  | **Non-experimental** | Non-experimental (quasi-experiment, descriptive and regression based study) |
|  | **Qualitative** | Case study |
|  |  | Other qualitative |
|  | **Modeling studies** | Modeling studies |
|  | **Systematic review** | Systematic reviews |
|  | **Scoping reviews** | Scoping reviews |
| **Evaluation types** | Impact evaluation |  |
|  | Process evaluation |  |
|  | Formative evaluation |  |
|  | Summative evaluation |  |
| **Publication type** | Peer review article |  |
|  | Pre-print peer review article |  |
|  | Report |  |
|  | Conference paper |  |
|  | Working paper |  |
|  | Discussion paper |  |
|  | Dissertation |  |
|  | Protocol |  |
| **Year of publication** | 1990 |  |
|  | 1991 |  |
|  | 1992 |  |
|  | 1993 |  |
|  | 1994 |  |
|  | 1995 |  |
|  | 1996 |  |
|  | 1997 |  |
|  | 1998 |  |
|  | 1999 |  |
|  | 2000 |  |
|  | 2001 |  |
|  | 2002 |  |
|  | 2003 |  |
|  | 2004 |  |
|  | 2005 |  |
|  | 2006 |  |
|  | 2007 |  |
|  | 2008 |  |
|  | 2009 |  |
|  | 2010 |  |
|  | 2011 |  |
|  | 2012 |  |
|  | 2013 |  |
|  | 2014 |  |
|  | 2015 |  |
|  | 2016 |  |
|  | 2017 |  |
|  | 2018 |  |
|  | 2019 |  |
|  | 2020 |  |
|  | 2021 |  |
|  | 2022 |  |
|  | 2023 |  |
